# ProteinTracker: an application for managing protein production and purification

**DOI:** 10.1186/1756-0500-5-224

**Published:** 2012-05-10

**Authors:** Stefan C Ponko, David Bienvenue

**Affiliations:** 1 Department of Biomedical Informatics and Medical Education, University of Washington, 850 Republican, Seattle, WA, 98109, USA; 2 VLST Inc, 307 Westlake Avenue North, Seattle, WA, 98109, USA

**Keywords:** Protein, Production, Purification, Reagent, Tracking, Prioritization, Web, Application

## Abstract

**Background:**

Laboratories that produce protein reagents for research and development face the challenge of deciding whether to track batch-related data using simple file based storage mechanisms (e.g. spreadsheets and notebooks), or commit the time and effort to install, configure and maintain a more complex laboratory information management system (LIMS). Managing reagent data stored in files is challenging because files are often copied, moved, and reformatted. Furthermore, there is no simple way to query the data if/when questions arise. Commercial LIMS often include additional modules that may be paid for but not actually used, and often require software expertise to truly customize them for a given environment.

**Findings:**

This web-application allows small to medium-sized protein production groups to track data related to plasmid DNA, conditioned media samples (supes), cell lines used for expression, and purified protein information, including method of purification and quality control results. In addition, a request system was added that includes a means of prioritizing requests to help manage the high demand of protein production resources at most organizations. ProteinTracker makes extensive use of existing open-source libraries and is designed to track essential data related to the production and purification of proteins.

**Conclusions:**

ProteinTracker is an open-source web-based application that provides organizations with the ability to track key data involved in the production and purification of proteins and may be modified to meet the specific needs of an organization. The source code and database setup script can be downloaded from http://sourceforge.net/projects/proteintracker. This site also contains installation instructions and a user guide. A demonstration version of the application can be viewed at http://www.proteintracker.org.

## Findings

### Background and purpose

A challenge for any organization that produces protein reagents is tracking batch information in a format that is easily accessible to all users. Laboratory notebooks (traditional paper or electronic) should serve as the primary repository of that information. However, due to the multiple steps involved in generating a single purified protein (molecular biology, cell culture, purification), the batch data often resides in multiple places. This makes it difficult to access all of the relevant information quickly. Over time, even a relatively small group of 5–10 researchers can generate hundreds, if not thousands of pieces of information that may be used by internal or external collaborators. The current high-throughput screening approaches that are employed by proteomics labs magnify this issue.

One solution is to capture important batch information into some form of spreadsheet, which has obvious limitations in versioning, ability to query the data, and reporting. Spreadsheets typically become the intermediate step before moving towards a commercial LIMS solution, if in-house expertise is available, or external resources are brought in to customize the LIMS. Regardless of the avenue that is selected, instituting a system for tracking this information early in the organization’s history is essential, as it only becomes more difficult as the size and complexity of the data set grows.

ProteinTracker provides smaller organizations with a LIMS solution that focuses solely on the key data required for protein production and purification, and therefore should not require the same level of site-specific customization as more complex commercial LIMS solutions. The most useful aspects of implementing ProteinTracker are traceability and transparency. The end users of the protein reagents have a wealth of information at their disposal without having to search through notebooks of 2–3 individuals to get the complete details of a particular batch. Users can make their own assessment of the batch quality. Changes in protein production methods are easily related to the activity, purity or yield of the final prep, since the “chain of custody” information from expression vector, conditioned media and purification are all linked and accessible.

Additionally, ProteinTracker has been particularly useful in facilitating the work performed with external contract organizations and other collaborators. By design, samples (expression constructs, purified proteins, etc.) are assigned a unique identifier that can be used as a means of easily identifying and tracking these samples at external sites. In one situation, nearly one thousand samples of conditioned media were sent to a collaborator, and a report summarizing the information on these samples was quickly generated using the data captured in ProteinTracker. In the case of a therapeutic protein being developed for human clinical trials, ProteinTracker was used to organize nearly a hundred different expression vectors that were generated for this program, which included multiple variations of the lead molecule and associated controls for *in vitro* and *in vivo* testing. For small organizations or academic groups involved in the preclinical development of protein therapeutics, ProteinTracker can be used as an intermediate form of data organization that is typically provided by formal quality control and quality assurance groups at larger companies.

The application was primarily designed to manage the data associated with protein reagent production, but not the experimental data generated with these reagents. However, it may potentially integrate with, or complement pre-existing workflow systems that do capture experimental data.

One enhancement made to ProteinTracker was the addition of a reagent request and prioritization feature. This proved to be very useful as a means of managing the work flow within the reagent production group, as well as keeping ‘customers’ informed of the status of the proteins they had requested. This facilitated the planning of experiments, while minimizing the amount of time spent updating numerous lab staff on their particular proteins of interest.

ProteinTracker has been in continuous use at VLST for the past ~5 years and has become a valuable resource for both the reagent service group and the researchers that depend on it for key components for their experiments. The application currently manages over 9,000 records, consisting of numerous plasmids, conditioned media records, cell lines and protein batches. The extensive use of open-source libraries and the source code licensing allow academic and smaller industrial institutions the ability to use and further enhance the system free of charge for their own specific situation.

### Application design overview

**Figure 1:**
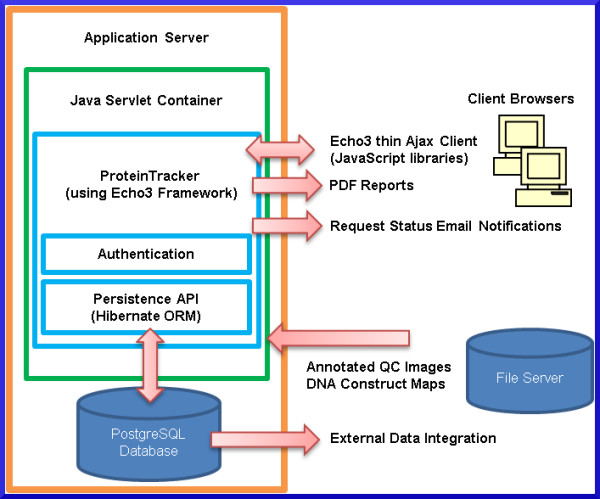
** High level schematic of ProteinTracker components.** ProteinTracker is a web application developed using the Echo3 open-source Ajax client–server framework that runs within a Java Servlet container. The presentation-tier consists of HTML and JavaScript rendered by server-side components. The logic-tier handles application logic including data management and validation, authentication, managing application errors and performing calculations. Data, with the exception of QC image files and DNA construct maps, are stored in a PostgreSQL relational database (data-tier). The open-source Hibernate Object Relational Management (ORM) library maps application data in the logic-tier to relational data in the data-tier.

ProteinTracker was developed using a three-tier client–server web application architecture consisting of a data tier, a logic tier and a presentation tier (Figure 1).

**Figure 2:**
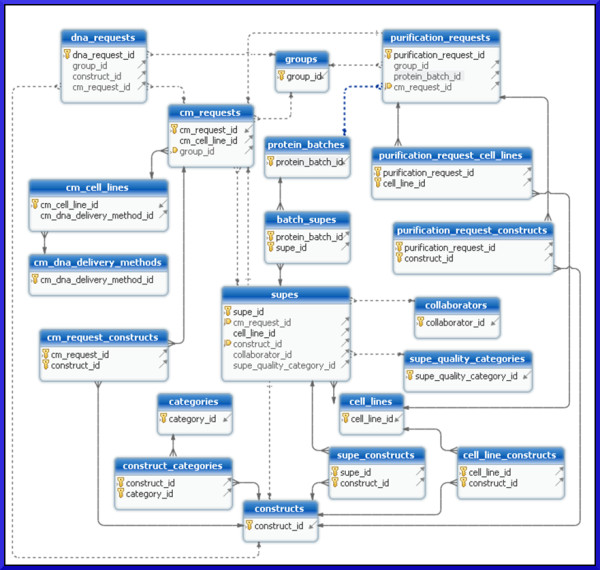
** Simplified database schema diagram.** This entity relationship diagram illustrates the relationships between the database tables. Only the primary and foreign key attributes are displayed to improve the readability and emphasize the relationships. Tables related to requests are near the top of the figure. The core data tables for tracking protein batches, supes, cell lines and DNA constructs are near the center of the figure. The ‘cm_’ prefix indicates conditioned media (supe) related tables.

The data tier is managed primarily by a PostgreSQL [1] relational database and secondarily by a network file server. Nearly all application data are stored in the database, with the exception of quality control (QC) gel images and DNA construct map files that are created and managed by proprietary commercial software. The QC gel images are stored on a network file server accessible to lab staff. The proprietary file-based DNA construct database used by the commercial software also resides on this file server, although this could potentially be located on a different file server. The DNA construct files are kept on the file server due to the inability of the commercial software to save and load files from a non-proprietary database. The software distribution includes a script that will create the complete database schema (Figure 2) and user account required by the application.

The web-based presentation layer was designed to emulate the look and feel of a desktop application and therefore does not use page-based navigation. Instead, the presentation layer consists of HTML and Ajax-enabled JavaScript rendered by the server-side open-source Echo3 framework components [2]. These synchronize state between the client browser and the server. The necessary client components are downloaded to the browser when the application is accessed over the network from a server-side Java Servlet [3]. Browser events generated by user interactions with the application are sent back to the server for processing by the logic-tier. The framework contains a number of standard user-interface components that accelerate the development of a user-interface, and allow for simplified user-interface design changes by utilizing common style sheets.

The logic-tier consists of Java-based object components running within the Echo3 framework. These components receive client-browser events and process them to save, update, load, or delete model objects to and from the database, send events back to the client for updating the screen, send email notifications, validate data, authenticate users, handle application errors, and perform calculations. Relational database persistence is provided using the open-source Hibernate framework [4]. The Hibernate framework maps object components to database tables using either annotations, or XML configuration files, and supports caching and an object-based query language (HQL) that is modelled after SQL. HQL supports querying relational data using the logic-tier objects, which removes the need to keep explicit database entity references in the logic-tier.

External applications may integrate with data in ProteinTracker at either the database level, or through the external links supported by ProteinTracker that allow other applications to link directly to either reagent or request records. The link format is described in the user documentation.

### Entering reagent data

**Figure 3:**
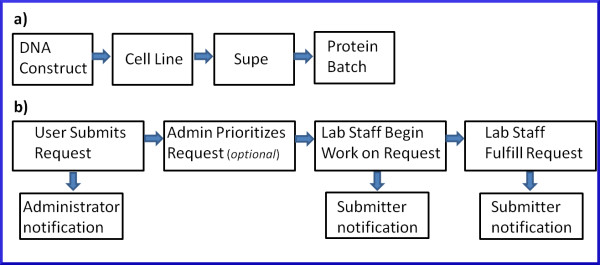
** Process flow diagram.a**). High level block diagram indicating the primary types of reagent data entered in the system and the order from left to right, in which they are typically entered. **b**). Block diagram indicating the basic operations and status changes for submission of requests for transient or stable expression, production of plasmid DNA, or delivery of aliquots from an existing batch of purified protein. Notification is in the form of an email with an embedded link to the originating request.

At a high level, users interact with ProteinTracker in one of two ways. The first is by entering, updating and querying reagent records including DNA constructs, cell lines, conditioned media samples (supes) and batches of purified protein (Figure 3a). The second is by entering, editing and prioritizing requests for reagents including DNA, supes and either new or existing batches of purified protein (Figure 3b).

**Figure 4:**
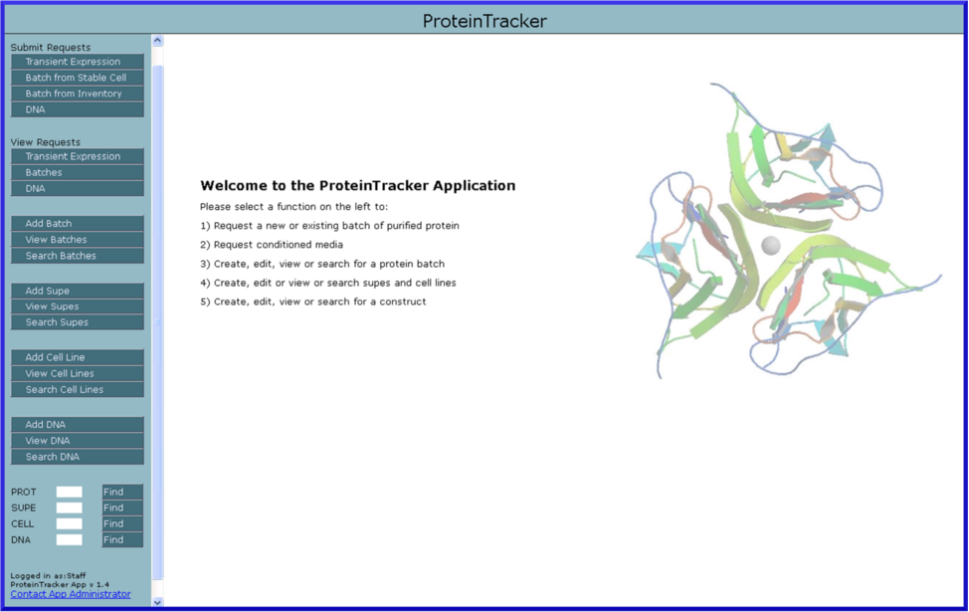
** Main application screen.** Application navigation options and quick-search fields are shown in this screenshot of the splash screen.

**Figure 5:**
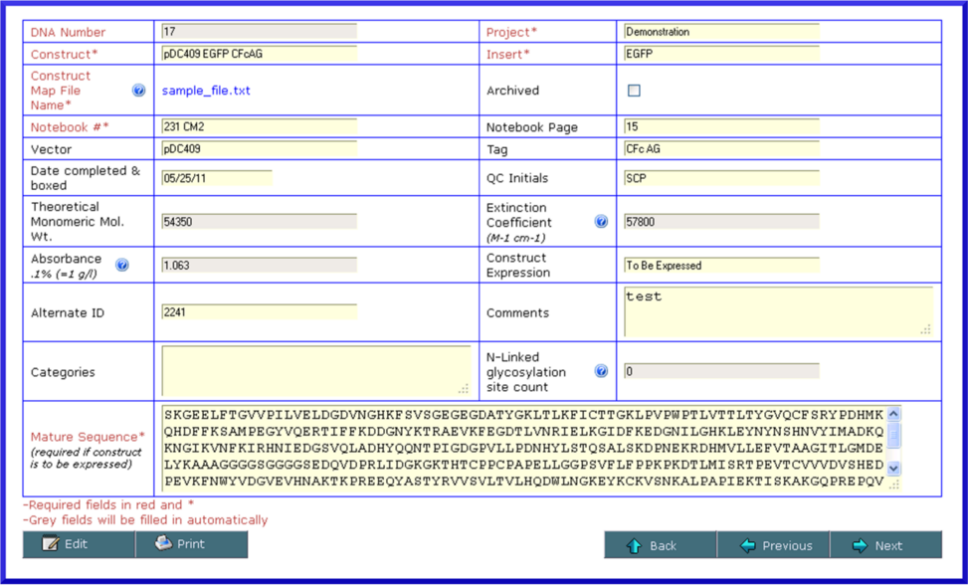
** Screenshot of DNA construct details.** Data entry typically begins with submission of a DNA construct. This screen captures information such as the mature protein amino acid sequence, construct map file produced by an external application, insert, tag, vector, notebook references, project name and several fields whose values are automatically calculated based on the mature sequence.

The panel on the left-hand side of the application contains the main navigation links for managing requests and records, while the right hand panel is updated dynamically depending on the selection (Figure 4). Request-related links are located at the top of the navigation panel, record management links in the middle, and quick navigation fields are at the bottom of the panel. Entering a record ID in one of these fields opens the appropriate record in the right panel. By default, all users of the application have read-only access to all records; however, users must log in using one of several administrator roles to add or edit any records as well as to prioritize requests. These roles and their configuration are specified in the application documentation. Typically, data entry begins with the submission of one or more DNA construct records (Figure 5). Users are required to enter a mature sequence, from which the theoretical monomeric molecular weight, absorbance, extinction coefficient, and number of N-linked glycosylation sites are calculated during record submission. Users can select on-line help to see how the calculations are performed. Additional data fields include the construct, project, vector, affinity purification tag and insert names, and notebook reference. A field is provided for entry of a file name associated with a separate construct map file. On-line help is also available on the submission screen so that the submitter knows where to store the construct map file. During record submission, the application will verify that the file reference is valid. If not, the user is notified that the file was not found. The network path to the construct map file directory is specified as part of the application configuration.

Users can view previously entered construct records by selecting the ‘View DNA’ link in the navigation panel. This displays a sortable, paged table display of all construct records. The page size is selectable and clicking on any construct record opens the record. It is also possible to page through the construct records one-by-one by selecting the ‘Previous’ and ‘Next’ buttons while viewing individual construct records. This holds true for cell line, supe and protein batch records as well. Selecting the ‘Search DNA’ link displays a search screen that exposes most of the construct attributes for searching. Search results are displayed using the same table format as that used by the ‘View DNA’ option.

A PDF report of the construct details can be displayed, saved, and printed using the print button displayed on the construct record. The cell line, supe and protein batch records are also printable, with the exception that when printing supes and protein batches, any related cell lines and constructs used to generate those reagents are included in the report.

**Figure 6:**
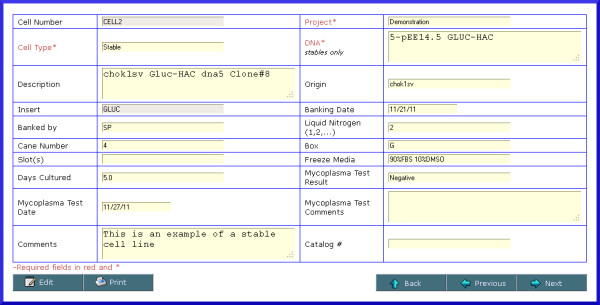
** Screenshot of cell line details.** The cell line data entry screen captures and displays information related to the cell line, including whether it is a parental or stable line (and DNA construct used for the latter), storage location information, mycoplasma test results, and cell line origin. The insert field is populated automatically for stable cell lines, depending on the selected DNA construct.

Cell lines are entered in a similar manner as that for constructs (Figure 6). When entering the record, the cell type must be specified. Cell types include stable, parental and hybridoma. When creating a stable cell line record, one or more (for co-transfections) DNA constructs previously entered must be selected. Additional fields are provided for tracking cell line origin and current freezer location, date that the cell line was banked, culture time, mycoplasma test results and any additional user annotations. Viewing and searching cell lines follows the same format as that for constructs.

**Figure 7:**
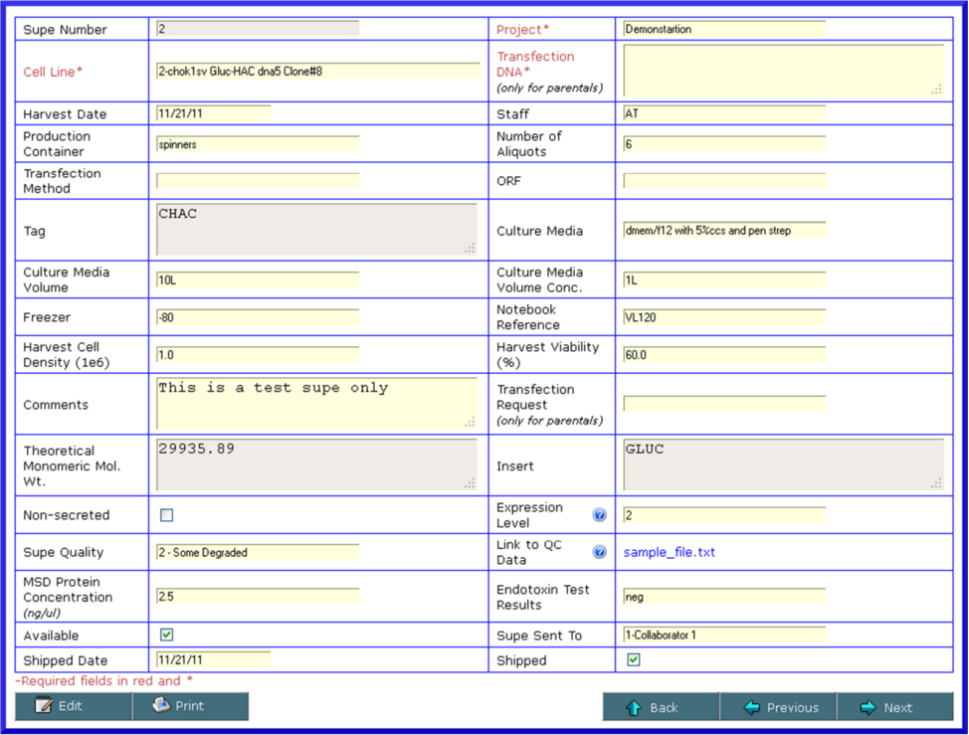
** Screenshot of supe details.** The supe screen captures and displays information related to the production of the protein supe such as the cell line (and transfection DNA in the case of transient transfections), number of aliquots, storage location, notebook reference, culture media information, endotoxin results, harvest information and several QC related fields. A field is also provided so that users may specify a link to a QC file associated with this supe.

Supe data entry begins with the selection of a specific cell line entered previously (Figure 7). To create an entry for a transient transfection, a parental cell line is selected, followed by one or more DNA constructs to be transfected. To instead create an entry for a stable transfection, one must select the appropriate stable cell line. Additional data fields support tracking of expression level, supe quality, links to Western blot images, endotoxin results, protein concentration, harvest related information, as well as remaining supe quantity and current location. Fields such as construct affinity tag, insert and molecular weight are populated based on the related DNA construct information. Viewing and searching supes follows the same format as that for cell lines and constructs.

**Figure 8:**
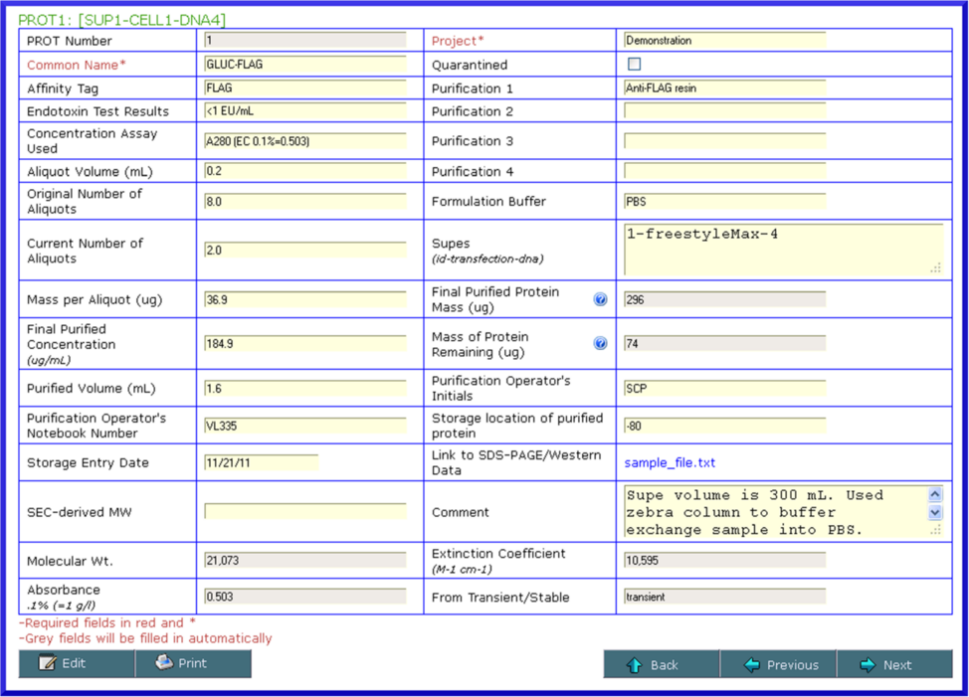
** Screenshot of protein batch details.** Information related to batches of purified protein include, among other items, the supe(s) that the protein was purified from, purification steps, endotoxin test results, volume, original and current number of aliquots, inventory information, as well as some basic protein characteristics calculated based on the theoretical amino acid sequence deduced from the DNA construct. An option is available to enter a link to a gel QC file. The relationship of the protein batch to the original supe, cell line and DNA construct are listed at the top left of the screen.

Once one or more supe records have been entered into the system, it is possible to select a supe for purification by selecting ‘Add Batch’ from the navigation panel (Figure 8). When creating a record for a new batch of purified protein a user may enter one or more purification steps, original and current number of aliquots, formulation buffer, protein concentration and purified volume and quality control information. In addition, a number of read-only fields are displayed based on the construct information that may be of value during the purification process, including the extinction coefficient, molecular weight and absorbance. The final purified protein mass and mass of remaining protein is calculated automatically during form submission.

### Requesting reagents

Users may submit a request for supe(s) to be generated from a transient transfection or stable cell line, request aliquots from an existing batch of purified protein, request a new batch of purified protein or request the production of plasmid DNA. Each option is available in the application navigation panel.

**Figure 9:**
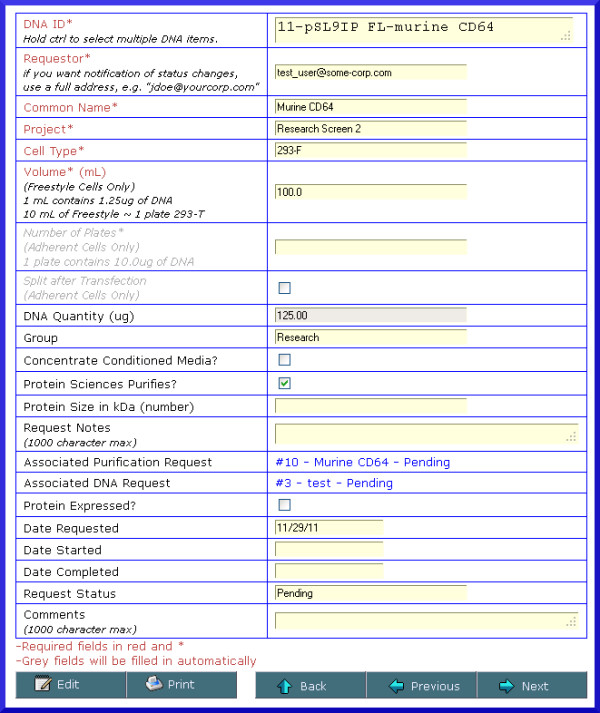
** Screenshot of a transfection request.** Users may submit a request for a transient transfection. As part of the submission process, they may also request that a DNA request and/or a purification request be automatically generated for (and associated with) this transfection request. If the request submitter specifies their email address in the Requestor field, they will be notified by email of any status changes to the request, including when work has begun on the request and when the request has been fulfilled.

When requesting supes from a transient expression (Figure 9), the request submitter selects a previously entered construct, a cell type and whether secondary requests should be submitted to the system for generating additional DNA or purification of the supe. When selecting the cell type, additional fields will be available to request the volume (for suspension cell culture), or the number of flasks (for cells grown adherently). A field is also provided to allow submitters to enter their email address to receive optional notification of all status changes to their request, including fulfillment of the request. When requests are initially submitted, a notification email is sent to all users that have been assigned to the request management group. The request screens for supes from stable cells lines or the production of DNA are self-explanatory. These request options also send notification emails to the submitter as the request status changes and is ultimately fulfilled.

Once it has been submitted into the system, a request may be viewed by selecting the appropriate type under ‘View Requests’. Each screen displays all requests that are Pending, Started or Fulfilled. All requests have a default status of ‘Pending’ after submission. Once lab staff begin to work on a request, they may log in and update the appropriate request status to ‘Started’. When all work for the request has been completed, the request status is updated to ‘Fulfilled’. Each status update will trigger an email to the request submitter notifying them of the status change that also provides a link to the request. Other fields in the requests may also be edited, and any request may be printed or saved as a PDF document.

**Figure 10:**
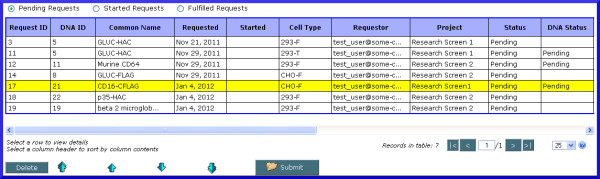
** Screenshot of request prioritization.** Requests for transfections, plasmid DNA, and batches of purified protein may be prioritized or deleted by an application administrator. Once logged in, the administrator may move requests up or down in the queue to reflect the priority of the request, by selecting the request and moving it towards the top of the queue for higher priority requests, or down for lower-priority requests.

An administrator can prioritize requests by clicking on the ‘Prioritize’ button at the bottom of the table view of requests, selecting a request and using the arrow keys to move it up and down in the request queue to change its relative priority (Figure 10). This option is only available when viewing requests for which work has not yet begun (i.e. in a ‘Pending’ state).

### Functionality comparison and performance

ProteinTracker has some similarities to other open-source LIMS such as PiMS [5] and OpenFreezer [6] that support reagent and workflow tracking; however, it focuses specifically on the most critical data that is generated in the course of protein reagent production. It also adds the ability for users to request reagents and be notified of status changes for requested reagents. ProteinTracker does not manage experimental data or workflow around such data. Smaller laboratories with limited staff and/or funding may find it difficult to invest the time or resources in configuring and supporting a more complex LIMS that supports workflow management for experimental data. This is particularly true for smaller laboratories that happen to have multiple, or rapidly changing workflows. ProteinTracker therefore represents an intermediate solution that lies between tracking reagent data manually and a more complex LIMS that may require significant customization.

ProteinTracker runs as a web application within Apache Tomcat [7] or any Servlet container supporting Java Servlet specification 2.4, and requires the installation of a PostgreSQL database. Installation from the source code distribution involves initial editing of the configuration files, compilation, and deployment to the Servlet container as a Web Application Archive (WAR) file. Per session memory consumption is minimal but increases temporarily during marshalling of data for presentation to the client tier. Memory settings for an installation will depend on the maximum number of concurrent number of users and amount of data accessed; however, a heap size setting of 1–2 GB for the Java virtual machine should be adequate for most installations. Smaller laboratories may require significantly less. For example, the public demonstration web site uses a heap size of 64 MB.

### Application development and testing

Testing of the application was performed using a combination of unit, functional, and user acceptance tests. A regression suite that includes the unit and functional test code written using the JUnit [8] test framework is included in the source distribution. The unit and functional test code verify that model objects, calculations, database access, authentication, and various screen components perform as expected when the application is modified.

The application was designed in discrete stages with each stage designed to manage one additional type of protein production-related reagent e.g. constructs, supes, cell lines, etc. At the completion of each stage, application users were given a chance to fully test the application in a test environment, with data cloned from the production database, to confirm that the functionality matched expectations before deploying updates to the production environment. No application changes were made without final user and management acceptance. The application was developed early during the companies ramping-up of protein production, so some existing data had to be backfilled and relational integrity established as each stage was developed. The benefit was that users were able to make use of the application early during the development-test-release cycle and were therefore more engaged in testing and providing feedback on desired functionality.

### Intended use

This application is to be used by service groups tasked with generating DNA or protein-related research reagents to support preclinical research programs. This provides the users of these reagents with a structure for both requesting new materials as well as accessing all the pertinent information relating to the manufacture of existing reagents.

### Future development

At this time, no further development is planned. The open-source licensing allows other users to download the application source code and libraries, and modify or further enhance the source code for their own specific work flows.

## Availability and requirements

The source code for ProteinTracker is open-source and freely available. Source code, installation instructions and a user manual are provided on the project home page listed below. Installation instructions and licensing information are also provided as part of the source code download.

**Project name:** ProteinTracker

**Project home page:**http://www.proteintracker.org

**Operating system(s):** Platform independent

**Programming language:** Java

**Other requirements:** Apache Tomcat or similar application server supporting Servlet specification 2.4, Java 1.6, PostgreSQL 8.3 or higher, and Apache Ant 1.7 [9] for compilation.

**License:** GNU LGPL v3.0 [10]

**Any restrictions to use by non-academics:** none

### Availability of supporting data

The data sets supporting the results of this article are included within the article (and its Additional file 1). The data sets supporting the results of this article are also available in the SourceForge repository, http://sourceforge.net/projects/proteintracker.

## Abbreviations

DNA, Deoxyribonucleic acid; HQL, Hibernate Query Language; HTML, HyperText Markup Language; JAR, Java Archive; LIMS, Laboratory Information Management System; ORM, Object Relational Mapping; PDF, Portable Document Format; QC, Quality Control; SQL, Structured Query Language; WAR, Web Application Archive.

## Competing interests

The authors declare they have no competing interests.

## Authors’ contributions

SP designed and developed the application using requirements and additional testing support provided by DB and team members of the Protein Sciences group at VLST. Both authors read and approved the final manuscript.

## Supplementary Material

Additional file 1 **Source Code Distribution.** ProteinTracker source code. Source code and build instructions for ProteinTracker. Click here for file

## References

[B1] PostgreSQL http://www.postgresql.org

[B2] Echo Web Framework http://echo.nextapp.com/site/echo3

[B3] Java http://www.oracle.com/technetwork/java

[B4] Hibernate http://www.hibernate.com

[B5] Morris C Pajon A Griffiths SL Daniel E Savitsky M Lin B Diprose JM da Silva AW Pilicheva K Troshin P van Niekerk J Isaacs N Naismith J Nave C Blake R Wilson KS Stuart DI Henrick K Esnouf RM The Protein Information Management System (PiMS): a generic tool for any structural biology research laboratory Acta Crystallogr D Biol Crystallogr 2011 D67 249 260 21460443 10.1107/S0907444911007943 PMC3069740

[B6] Olhovsky M Williton K Dai AY Pasculescu A Lee JP Goudreault M Wells CD Park JG Gingras AC Linding R Pawson T Colwill K OpenFreezer: a reagent information management software system Nat Methods 2011 8 612 613 10.1038/nmeth.1658 21799493

[B7] Apache Tomcat http://tomcat.apache.org

[B8] JUnit http://junit.sourceforge.net

[B9] Apache Ant http://ant.apache.org

[B10] GNU Lesser Public License http://www.gnu.org/licenses/lgpl.html

