# Supplementary material for: ProteinTracker: an application for managing protein production and purification
Source: BMC Res Notes. 2012 May 10;5:224. doi: 10.1186/1756-0500-5-224 (PMC3436699; doi:10.1186/1756-0500-5-224)
Supplement: Additional file 1 — Source Code Distribution. ProteinTracker source code. Source code and build instructions for ProteinTracker. [file 1756-0500-5-224-S1.zip › proteintracker/web/fileNotFound.jsp]

 File Not Found
<%
String filePath = request.getParameter("filePath");
String fileName = request.getParameter("fileName");
String fullFileName = filePath + fileName;
%>

## File Not Found: <%= fullFileName %>

The file listed above could not be found.   
If you are certain that this file exists, and that the link should have worked,
please contact your applications system administrator.
